# Activation of the PI3K/Akt/mTOR/p70S6K Pathway is Involved in S100A4-Induced Viability and Migration in Colorectal Cancer Cells: Erratum

**DOI:** 10.7150/ijms.69070

**Published:** 2022-01-21

**Authors:** Haiyan Wang, Liang Duan, Zhengyu Zou, Huan Li, Shimei Yuan, Xian Chen, Yunyuan Zhang, Xueru Li, Hui Sun, He Zha, Yan Zhang, Lan Zhou

**Affiliations:** 1 Key Laboratory of Laboratory Medical Diagnostics, Ministry of Education, Department of Laboratory Medicine, Chongqing Medical University, Chongqing 400016, China;; 2 Department of Laboratory, the First People's Hospital of Jiulongpo District, Chongqing 400050, China.

The images of original Figure 4B were incorrectly assembled. All authors were informed and approved the corrected figures.

In our paper 1, Figure 4B should be corrected as follows.

## Figures and Tables

**Figure 4:**
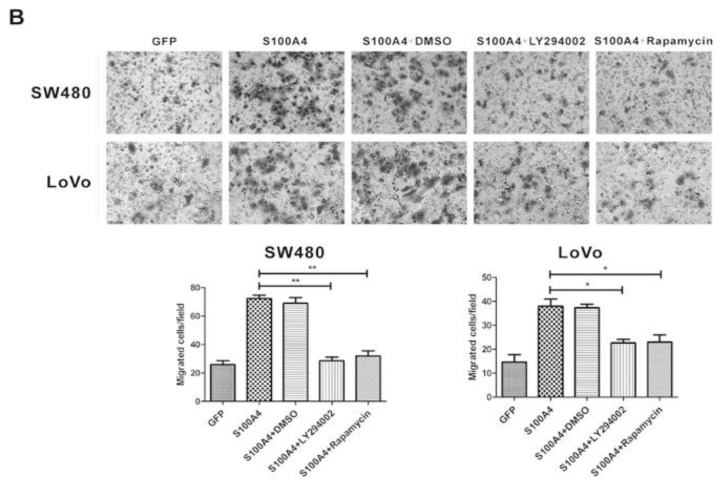
LY294002 and rapamycin suppress S100A4-induced viability and migration of CRC cells. (A) Effects of L Y294002 and rapamycin on S100A4-induced viability of SW480 and LoVo cells were detected by MTT assay. *P<0.05 vs S100A4 group. (B) Effects of LY294002 and rapamycin on S100A4-induced migration of SW480 and LoVo cells were detected by transwell assay, ×200. *P<0.05, **P<0.01 vs S100A4 group.
